# Nano‐LYTACs for Degradation of Membrane Proteins and Inhibition of CD24/Siglec‐10 Signaling Pathway

**DOI:** 10.1002/advs.202300288

**Published:** 2023-03-03

**Authors:** Kun Wang, Albert Yu, Kewei Liu, Chunyan Feng, Yibo Hou, Jiawei Chen, Shaohua Ma, Laiqiang Huang, Xiaoyong Dai

**Affiliations:** ^1^ Institute of Biopharmaceutical and Health Engineering Shenzhen Key Laboratory of Gene and Antibody Therapy State Key Laboratory of Chemical Oncogenomics Shenzhen International Graduate School Tsinghua University Shenzhen Guangdong 518055 China; ^2^ School of Life Sciences Tsinghua University Beijing 100084 China

**Keywords:** ASGPR, CD24, GOx, liver cancer, lysosomal degradation, LYTACs

## Abstract

Lysosome‐targeting chimeras (LYTACs) are an emerging therapeutic modality that effectively degrade cancer cell membranes and extracellular target proteins. In this study, a nanosphere‐based LYTAC degradation system is developed. The amphiphilic peptide‐modified *N*‐acetylgalactosamine (GalNAc) can self‐assemble into nanospheres with a strong affinity for asialoglycoprotein receptor targets. They can degrade different membranes and extracellular proteins by linking with the relevant antibodies. CD24, a heavily glycosylated glycosylphosphatidylinositol‐anchored surface protein, interacts with Siglec‐10 to modulate the tumor immune response. The novel Nanosphere‐AntiCD24, synthesized by linking nanospheres with CD24 antibody, accurately regulates the degradation of CD24 protein and partially restores the phagocytic function of macrophages toward tumor cells by blocking the CD24/Siglec‐10 signaling pathway. When Nanosphere‐AntiCD24 is combined with glucose oxidase, an enzyme promoting the oxidative decomposition of glucose, the combination not only effectively restores the function of macrophages in vitro but also suppresses tumor growth in xenograft mouse models without detectable toxicity to normal tissues. The results indicate that GalNAc‐modified nanospheres, as a part of LYTACs, can be successfully internalized and are an effective drug‐loading platform and a modular degradation strategy for the lysosomal degradation of cell membrane and extracellular proteins, which can be broadly applied in the fields of biochemistry and tumor therapeutics.

## Graphical abstract

Lysosome‐targeting chimeras are an emerging therapeutic modality that effectively degrade cancer cell membranes and extracellular target proteins. This study develops a nanosphere‐based lysosome‐targeting chimera degradation system, which can be successfully internalized and is an effective drug‐loading platform and a modular degradation strategy for the lysosomal degradation of cell membrane and extracellular proteins.

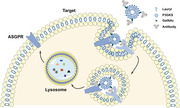

## 1 Introduction

Compared with traditional small‐molecule therapy, targeted protein degradation has obvious advantages and has become a powerful treatment method for handling undruggable targets.^[^ ^1^, ^2^ ^]^ Unlike proteolysis‐targeting chimeras,^[^ ^3^, ^4^ ^]^ lysosome‐targeting chimeras (LYTACs) degrade extracellular and membrane‐bound proteins (POI) through the lysosome degradation pathway.^[^ ^5^, ^6^ ^]^ LYTACs are small molecules with dual affinity formed by connecting a POI‐binding element with a lysosome‐shuttling receptor ligand, such as cation‐independent mannose 6‐phosphate receptor (CI‐M6PR)^[^ ^7^ ^]^ and asialoglycoprotein receptor (ASGPR).^[^ ^8^, ^9^ ^]^ ASGPR is a cell membrane receptor specifically expressed by mammalian hepatic cells.^[^ ^10^ ^]^ ASGPR mediates the binding, internalization, and lysosomal clearance of glycoproteins containing terminal galactose or *N*‐acetylgalactosamine residues (asialoglycoproteins) from circulation.^[^ ^11^, ^12^, ^13^ ^]^ In addition, ASGPR belongs to the recycling receptor group and undergoes constitutive endocytosis and recycling with or without ligands, making it a reliable and effective target for protein degradation research and treatment of liver cancer.^[^ ^14^, ^15^, ^16^, ^17^ ^]^ Ever since Banik et al. designed and developed the first LYTACs in 2020, an increasing number of researchers have begun to pay attention to this novel cancer treatment method. LYTACs have been gradually applied to various cancers, including liver and breast cancers, where they have achieved good therapeutic effects, significantly reducing the expression of cancer‐related proteins.^[^ ^18^ ^]^

CD24 is a mucin‐like glycosylphosphatidylinositol‐anchored surface protein widely expressed in several solid tumors and has recently gained attention.^[^ ^19^, ^20^ ^]^ Studies have shown that CD24 is highly expressed in hepatocellular carcinoma (HCC) tissues,^[^ ^21^, ^22^ ^]^ and its expression is closely related to the proliferation, migration, and invasion of cancer cells.^[^ ^23^, ^24^ ^]^ CD24 also plays an important role in regulating tumor immune response.^[^ ^25^, ^26^ ^]^ Another regulator of tumor immune response is Siglec‐10, an innate immune checkpoint that inhibits the activation of immune cells and is overexpressed in macrophages. They both protect cancer cells from being “eaten” by macrophages through the CD24/Siglec‐10 signaling pathway.^[^ ^27^, ^28^ ^]^ Hence, blocking the CD24/Siglec‐10 signaling pathway is of great value in enhancing the immune function of macrophages. Currently, treatment targeting CD24 mainly includes three methods: monoclonal antibody, antibody‐drug conjugates, and Chimeric Antigen Receptor T‐cell therapy.^[^ ^29^, ^30^ ^]^ However, these methods do not change the expression of CD24 and only achieve transient signal inhibition. Therefore, there is an urgent need to develop new methods to treat cancer by altering the expression of CD24.

In recent years, starvation therapy has emerged as a promising cancer therapy strategy that suppresses tumor growth by depriving essential nutrients.^[^ ^31^ ^]^ Glucose oxidase (GOx) catalyzes glucose to hydrogen peroxide (H_2_O_2_) and accelerates glucose utilization.^[^ ^32^, ^33^ ^]^ However, starvation therapies based on GOx are restricted by a lack of targeting.^[^ ^34^, ^35^, ^36^ ^]^ Combining LYTACs with GOx can not only remedy the lack of accurate targeting but also enhance the effect of GOx in cancer starvation therapy.^[^ ^37^, ^38^, ^39^ ^]^

In this study, we used polypeptide‐modified *N*‐acetylgalactosamine self‐assembly to form nanospheres that could bind to ASGPR. The excess amino groups on the nanospheres ensured the subsequent linking of the antibody. Additionally, the internal hydrophobic structure of the nanospheres provided space for loading GOx. More importantly, the purified high‐concentration CD24 antibody was successfully linked to the nanospheres by crosslinking, forming novel LYTACs with a CD24 degradation function termed “Nanosphere‐AntiCD24”. The Nanosphere‐AntiCD24 could be selectively internalized by CD24‐overexpressed HCC cells and subsequently transported CD24 protein on the cell membrane to the lysosome for degradation. The degradation of CD24 could then lead to the weakening of macrophage immunosuppression regulated by the CD24/Siglec‐10 signaling pathway. Finally, “Nanosphere‐AntiCD24” was loaded with GOx, and the targeted release of GOx continuously depleted endogenous glucose in HCC cells, inducing starvation therapy. This strategy demonstrated a satisfactory synergistic therapeutic effect against HCC in both in vitro and in vivo experiments.

## 2 Results

### 2.1 Construction and Characterization of ASGPR‐Targeted Nanoparticles

**Figure 1:**
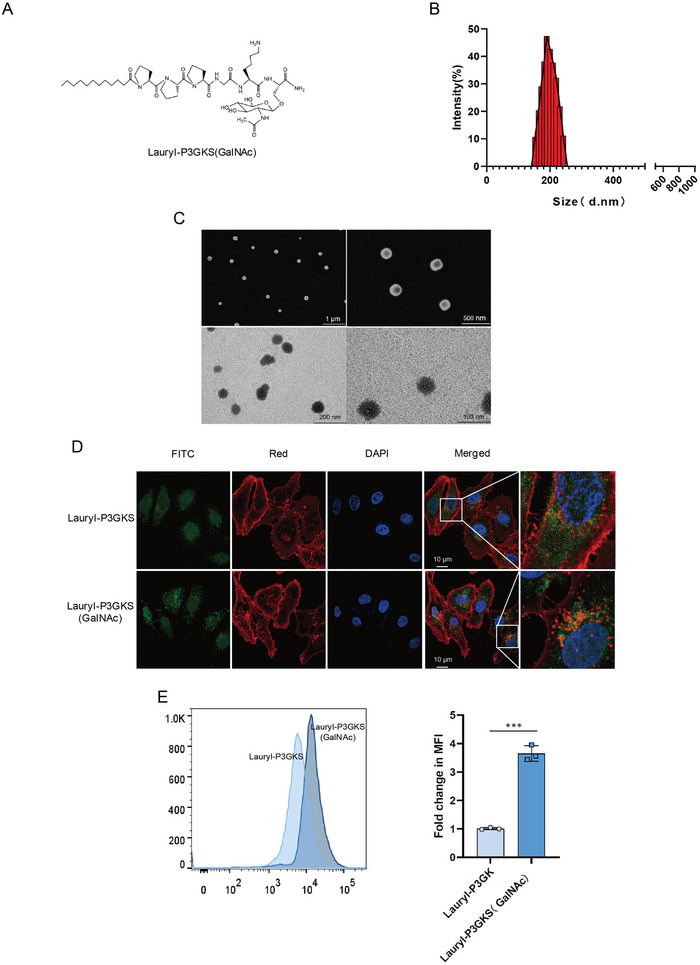
Construction and characterization of ASGPR‐targeted nanoparticles. A) Structure of amphiphilic glycopeptides, Lauryl‐P3GKS (GalNAc). B) Particle size distribution patterns of glycopeptide nanoparticles. C) SEM and TEM micrographs of glycopeptides nanoparticles. D) Localization of glycopeptides nanoparticles (green) was imaged with fluorescent confocal microscopy. Cell nuclei were stained by DAPI (blue) and the membrane was stained by red dye. E) The fluorescence intensity analysis of glycopeptides nanoparticles.

We intended to use peptide ligands for ASGPR to develop LYTACs and improve their binding abilities. Solid‐phase peptide synthesis was used to create amphiphilic glycopeptides, Lauryl‐P3GKS (*N*‐acetylgalactosamine (GalNAc)) (**Figure** **1**A), which were then analyzed by liquid chromatography‐mass spectrometry (LC‐MS, Figure S1, Supporting Information). A hydrophilic lysine residue in the peptide sequence serves as an efficient location for crosslinking, and modified GalNAc ensures that the peptide specifically binds to ASGPR. Amphiphilic peptides self‐assembled into stable peptide nanoparticles in an aqueous solution, and unmodified peptides were used as a control. The Zetasizer showed that the nanoparticles were uniformly dispersed and had a size distribution of ≈200 nm (Figure 1B). Scanning electron microscopy (SEM) and transmission electron microscopy (TEM) were used to observe the morphologies of the nanostructures, and the results revealed that their shapes were uniform and regular, which was compatible with the findings of the particle size detector (Figure 1C). Using membrane dyes and fluorescence imaging, we analyzed endocytosis and found that modified GalNAc‐ and fluorescein isothiocyanate (FITC)‐labeled nanoparticles could bind to the cell membrane and enter HepG2 cells to produce additional green vesicles that overlapped the red membrane (Figure 1D). The overlapping fluorescence intensity was then analyzed by flow cytometry, and the statistical results showed that the fluorescence intensity was significantly increased in the Lauryl‐P3GKS (GalNAc) group (Figure 1E). These results suggest that ASGPR‐targeted nanospheres could bind to ASGPR and successfully translocate into cells.

### 2.2 ASGPR‐Driven LYTACs‐Mediated Membrane Proteins to Lysosomes for Degradation

**Figure 2:**
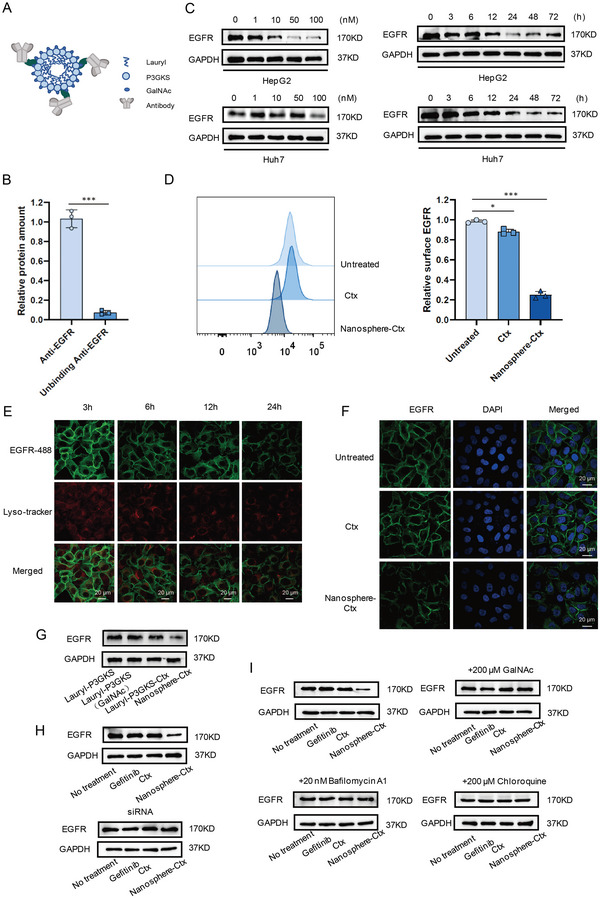
ASGPR‐driven LYTACs‐mediated membrane proteins to lysosomes for degradation. A) Structure of Nano‐LYTACs. B) Quantitative detection of Ctx protein before and after antibody and nanoparticles conjunction. C) Western blotting examination for EGFR in HepG2 and Huh7 cells treated with Nanosphere‐Ctx. D) Flow cytometry analysis of EGFR expression on HepG2 cell surface before and after Nanosphere‐Ctx treatment. E) Visualization of EGFR re‐localization in HepG2 cells by confocal microscopy after treatment with 50 × 10^−9^ m Ctx conjugate for 24 h. F) Visualization of EGFR degradation in HepG2 cells by confocal microscopy after treatment with 50 × 10^−9^ m Ctx conjugate for 24 h. G) EGFR levels in HepG2 cells treated with glycan‐modified nanosphere, antibody‐modified nanosphere, or Nano‐LYTAC. H) EGFR levels in HepG2 cells expressing a control siRNA targeting ASGPR after treatment with 50 × 10^−9^ m Nanosphere‐Ctx for 24 h. I) EGFR levels in HepG2 cells after treatment with 50 × 10^−9^ m Nanosphere‐Ctx for 24 h in the presence of 200 × 10^−6^ m GalNAc, 20 × 10^−9^ m BafilomycinA1, or 200 × 10^−6^ m chloroquine.

To demonstrate the specificity of ASGPR‐driven LYTACs, we constructed nonspecific nanoparticles using the lysine residue of the nanospheres to link cetuximab (Ctx), an epidermal growth factor receptor (EGFR)‐blocking antibody approved by the Food and Drug Administration (**Figure** **2**A). The peptides self‐assembled into nanospheres. Lysine on the nanospheres contains free amino groups that can react with the free carboxyl groups of antibody molecules through crosslinking. The nanospheres were connected to antibodies through the highly active crosslinking reaction reagents 1‐ethyl‐3‐[3‐dimethylaminopropyl] carbodiimide hydrochloride (EDC) and *N*‐hydroxysulfosuccinimide (Sulfo‐NHS). Through quantitative analysis, we determined the related protein content. The antibody concentration decreased rapidly after the reaction, indicating that the antibody was completely attached to the nanospheres (Figure 2B). Similarly, we calculated the number of antibodies connected to each nanosphere according to the formula, and the results showed that all three antibodies could attach to the nanospheres (Figure S2A, Supporting Information). Therefore, binding efficiency to the corresponding targets can be effectively improved. To investigate the degradation ability of the Ctx‐functionalized glycopolypeptide‐nanospheres (Nanosphere‐Ctx), HepG2 and Huh7 cells were treated. Following cell lysis, total EGFR levels were measured. With increasing Nanosphere‐Ctx concentration, EGFR protein levels exhibited a significant dose‐dependent decrease. Moreover, EGFR degradation was observed after treatment with 50 × 10^−9^ m Nanosphere‐Ctx for 6 h, peaked between 24 and 48 h, and was sustained for at least 72 h (Figure 2C). The flow cytometry results also confirmed that the EGFR protein level was significantly reduced by Nanosphere‐Ctx (Figure 2D and Figure S2B, Supporting Information). LYTAC‐mediated degradation of EGFR by Ctx was also observed by immunofluorescence microscopy. Immunofluorescence microscopy revealed a noticeable re‐localization of EGFR from the plasma membrane to the intracellular vesicles after treatment with Nanosphere‐Ctx compared to free Ctx (Figure 2E and Figure S2C, Supporting Information). Moreover, visualization and quantification of EGFR following Nanosphere‐Ctx treatment showed diminished membrane EGFR signals, supporting the flow cytometry and western blot findings (Figure 2F and Figure S2D, Supporting Information). We then tested the effect of glycan‐modified nanospheres, antibody‐modified nanospheres, and Nano‐LYTAC on EGFR degradation, and the results showed that only Nanosphere‐Ctx could promote EGFR protein degradation (Figure 2G). We also compared the degradation performance of Nanosphere‐Ctx to that of siRNA‐mediated ASGPR knockout hepatoma cells and found that EGFR degradation was more pronounced in HepG2 and Huh7 cells that expressed ASGPR normally (Figure 2H and Figure S2E, Supporting Information). Nanosphere‐Ctx degradation of EGFR was inhibited in the presence of exogenous GalNAc, while treatment of cells with Bafilomycin A1 or chloroquine prevented the degradation promoted by Nanosphere‐Ctx, indicating that Nanosphere‐Ctx was dependent on ASGPR binding and lysosomal acidification (Figure 2I and Figure S2F, Supporting Information). All experiments confirmed that GalNAc‐modified LYTACs effectively bound to the target protein and promoted its degradation through the lysosomal pathway.

### 2.3 CD24‐Targeted LYTACs Reinforced Degradation of CD24 Proteins

**Figure 3:**
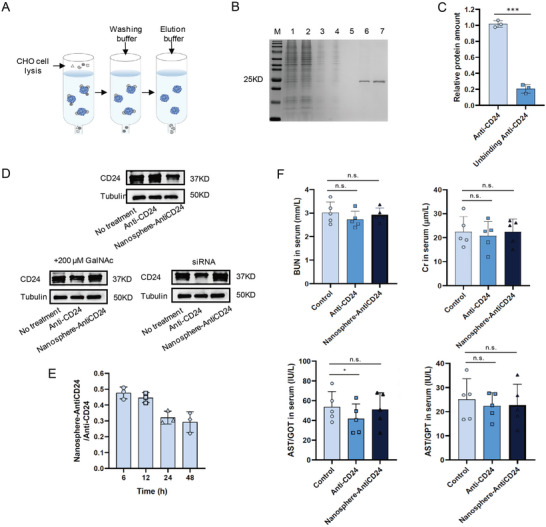
CD24‐targeted LYTACs reinforced degradation of CD24 proteins. A) The protocol of CD24 antibody purification. B) CD24 antibody was analyzed by SDS‐PAGE via Coomassie Blue staining (1: cell lysis, 2: unbinding protein, 3: washing 1, 4: washing 2, 5: washing 3, 6: elution 1, 7: elution 2). C) Quantitative detection of Anti‐CD24 protein before and after antibody and nanoparticles conjunction. D) CD24 protein levels after treatment with 100 × 10^−9^ m Nanosphere‐AntiCD24 for 24 h to HepG2 cells in the presence of 200 × 10^−6^ m GalNAc or HepG2 cells expressing a control siRNA targeting ASGPR. E) Quantification of serum Nanosphere‐AntiCD24 relative to Anti‐CD24 after intraperitoneal injection. F) Concentration of aspartic acid transferase (AST), creatinine, and blood urea nitrogen in serum of mice injected with Nanosphere‐AntiCD24.

The interaction between CD24 on cancer cells and Siglec‐10 on macrophages inhibits the phagocytosis of tumor cells by macrophages. Theoretically, the degradation of CD24 protein enhances the phagocytic function of macrophages to achieve a better therapeutic effect. Thus, we investigated whether linking other targeting antibodies to the nanospheres would affect the degradation of the corresponding target proteins. He et al. have shown that the CD24 antibody G7S has a high specific affinity for the CD24 protein of liver cancer cells,^[^ ^40^ ^]^ so we chose this sequence as the CD24 antibody sequence for the next experiment. We constructed a prokaryotic expression system for the expression of the CD24 antibody and purified the proteins using a protein purification system (**Figure** **3**A). To assess the results of antibody purification, we analyzed the purity of the samples at different purification stages and ultimately obtained a high‐purity CD24 antibody (Figure 3B). Subsequently, we constructed ASGPR‐driven LYTACs termed Nanosphere‐AntiCD24 and verified the successful linking of the CD24 antibodies onto the nanospheres through quantitative protein analysis (Figure 3C). HepG2 cells were stimulated with 100 × 10^−9^ m Nanosphere‐AntiCD24 for 24 h. The levels of CD24 on the cell membrane were tested, and the results showed that Nanosphere‐AntiCD24 accelerated the degradation of CD24 protein, which was inhibited by ASGPR knockout and exogenous GalNAc (Figure 3D). Based on these results, we examined how nanosphere conjugation affects antibody clearance in vivo. BALB/c mice were intraperitoneally injected with 10 mg kg^−1^ Anti‐CD24 or Nanosphere‐AntiCD24, and the serum antibody levels were assayed by his‐tag enzyme‐linked immune sorbent assay (ELISA). There was a moderate decrease in serum Nanosphere‐AntiCD24 levels (Figure 3E), and no notable toxicity was observed in the liver (Figure 3F). These results revealed that the synthesized Nanosphere‐AntiCD24 had better stability than the free antibody, and the effect on the liver was within the normal pharmacological range.

### 2.4 Nanosphere‐AntiCD24 Degrades CD24 Protein and Augments Phagocytosis of Macrophage by Blocking CD24/Siglec‐10 Signaling Pathway

**Figure 4:**
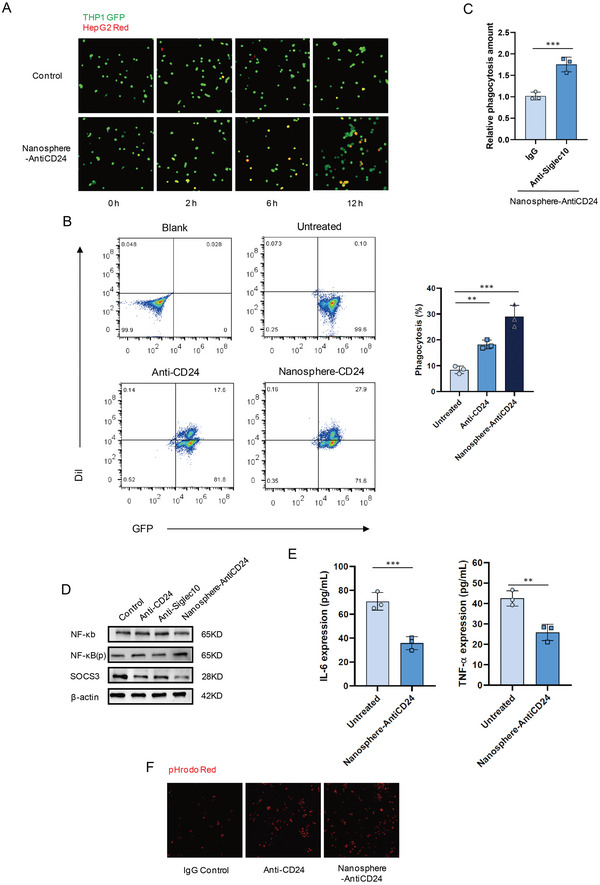
Nanosphere‐AntiCD24 degrades CD24 protein and augments the phagocytic activity of macrophage by blocking CD24/Siglec‐10 signaling pathway. A) Fluorescence microscopy images of the phagocytic activity of THP1 GFP^+^ cells on HepG2 Red^+^ cells treated with 100 × 10^−9^ m Nanosphere‐AntiCD24 for 24 h. B) Flow cytometry‐based measurement of phagocytosis of HepG2 cells (Red^+^) by co‐cultured THP1 GFP^+^ cells, in the presence of 100 × 10^−9^ m Anti‐CD24 or Nanosphere‐AntiCD24 for 24 h. C) Phagocytosis of HepG2 Red^+^ cells treated with 100 × 10^−9^ m Nanosphere‐AntiCD24 for 24 h in the presence of anti‐Siglec‐10 mAb or IgG control. D) Siglec‐10 downstream NF‐*κ*B, p‐NF‐*κ*B, and SOCS3 levels after treatment with 100 × 10^−9^ m Anti‐CD24, 100 × 10^−9^ m Anti‐Siglec10, or Nanosphere‐AntiCD24 for 24 h. E) Detection of Siglec‐10‐related cytokine secretion. F) Images from live‐cell microscopy phagocytosis assays of pHrodo‐red^+^ HepG2 cells treated with 100 × 10^−9^ m Anti‐CD24, 100 × 10^−9^ m Anti‐Siglec10, or Nanosphere‐AntiCD24 for 2 h.

In addition, we investigated whether the degradation of the CD24 protein in HCC cells affects tumor‐associated macrophages in the tumor microenvironment. HepG2, marked with a red fluorescent probe after being incubated with 100 × 10^−9^ m Nanosphere‐AntiCD24 for 24 h, was co‐cultured with GFP+ (M1‐like) THP1 cells. Fluorescence microscopy revealed greater phagocytic activity of the Nanosphere‐AntiCD24‐treated cells compared with the untreated cells (**Figure** **4**A and Figure S3A, B Supporting Information). A flow cytometry‐based phagocytosis assay revealed a robust increase in phagocytic activity upon the addition of Nanosphere‐AntiCD24 (Figure 4B). To further determine whether this increase in immunosuppression was achieved by disrupting the CD24/Siglec‐10 signaling pathway, a Siglec‐10 monoclonal antibody was used to block Siglec‐10 in THP1 cells, which could augment the phagocytosis of HepG2 cells by macrophages (Figure 4C). Additionally, we treated the HepG2 cells with Nanosphere‐AntiCD24 and co‐cultured with THP1 cells. The expression of genes downstream of Siglec‐10 was analyzed. We found that p‐NF‐*κ*B, an important transcription factor that induces gene expression, was significantly increased, while there was a lower expression of SOCS3, a negative regulator of cytokine signal transduction, after Nanosphere‐AntiCD24 treatment (Figure 4D). This confirmed that the targeted degradation of CD24 by Nanosphere‐AntiCD24 affected communication between macrophages and cancer cells through the CD24/Siglec‐10 signaling pathway. Co‐culturing M1‐like macrophages expressing Siglec‐10 with either WT or HepG2 cells, which had their CD24 protein degraded by Nanosphere‐AntiCD24, resulted in lowered levels of Siglec‐10 related cytokines, including interleukin 6 (IL‐6) and tumor necrosis factor alpha (TNF‐*α*). This suggested that Nanosphere‐AntiCD24 was sufficient to potentiate the secretion of IL‐6 and TNF‐*α*, which plays a considerable role in the activation of immune cells (Figure 4E). HepG2 cells were labeled with pHrodo Red, exhibit pH‐sensitive fluorescence emission, and its emission intensity increases with increasing acidity. pHrodo Red glucan is almost dark in the extracellular environment; however, after endocytosis, the acidic environment of the endosome causes the glucan conjugate to produce bright red fluorescence signals. Further studies demonstrated that HepG2 cells treated with Nanosphere‐AntiCD24, were more readily engulfed and degraded in the low‐pH phagolysosome (Figure 4F). This indicated that Nanosphere‐AntiCD24 degraded CD24 protein and regulated the phagocytic activity of macrophages by inhibiting the CD24/Siglec‐10 signaling pathway.

### 2.5 ASGPR‐Mediated LYTACs United with GOx Inhibit Tumor Growth In Vivo

**Figure 5:**
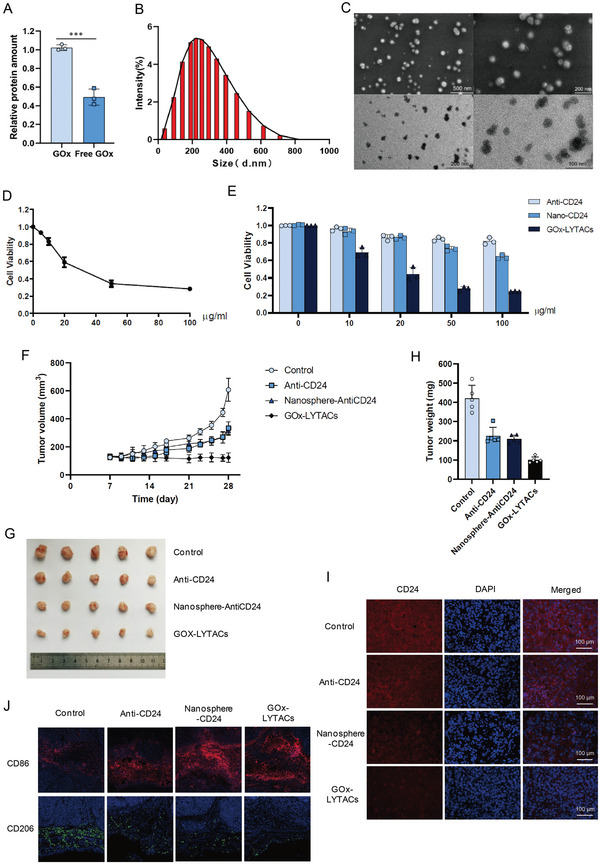
ASGPR‐mediated LYTACs combined with GOx inhibit tumor growth in vivo. A) Quantitative detection of GOx outside the hydrophobic nanoparticles. B) Particle size distribution of glycopeptide nanoparticles loaded with GOx. C) SEM and TEM images of glycopeptides nanoparticles loaded with GOx. D) Cell viability assay on cells treated with GOx‐LYTACs for 24 h performed using CCK‐8. E) Cell viability assay on cells treated with Anti‐CD24, Nanosphere‐AntiCD24, or GOx‐LYTACs for 24 h performed using CCK‐8. F) Tumor growth curve of HepG2 xenograft mice. G) Picture of dissected hepatoma tumor from mice 28 days after inoculation. H) Statistical diagram of tumor volume of experimental and control group HepG2 xenograft mice. I) Immunofluorescence of tumor samples, CD24 (red) and cell nuclei (blue). J) Immunofluorescence of tumor samples, CD86 (red) and CD206 (green).

To validate our GalNAc‐modified nanospheres, we investigated whether they could be developed as a useful drug‐loading platform. GOx was encapsulated in the hydrophobic center of the nanoparticles to inhibit tumor growth. The nanocomposites thus formed were subjected to quantitative analysis of the GOx protein to confirm GOx loading into the nanospheres (**Figure** **5**A). These had a uniform spherical geometry with an average size of ≈210 nm (Figure 5B). The obtained SEM and TEM images exhibited separate nanosphere structures, which was consistent with the results from the particle size analyzer (Figure 5C). To improve the antitumor effect, the CD24 antibody was linked to the nanocomposites forming GOx‐LYTACs. The inhibitory effects of GOx‐LYTACs were examined and compared using a standard cell counting kit‐8 (CCK‐8) assay. As shown in Figure 5D, 30 µg mL^−1^ GOx‐LYTACs inhibited the growth of HepG2 cells, and the greater the concentration of GOx‐LYTACs, the greater the inhibitory effect. Additionally, we compared cell viability, which revealed a declining trend with increasing GOx‐LYTAC treatment concentration (Figure 5E). The in vivo antitumor activity of GOx‐LYTACs was evaluated in a HepG2 xenograft mouse model. BALB/c mice were injected intraperitoneally with 100 µg kg^−1^ GOx‐LYTACs. Tumor growth in the mice was observed for 21 days (Figure 5F), and the tumor weight was measured (Figure 5G,H). The results confirmed that treatment with GOx‐LYTACs resulted in the most potent tumor inhibition without toxicity in vivo (Figure S4A–D, Supporting Information). Immunofluorescence staining was conducted for degradation analysis after euthanizing the mice. The red fluorescence intensity in the GOx‐LYTACs treatment group was significantly lower than that in the control group, suggesting that the degradation effect of GOx‐LYTACs was fully functional in vivo (Figure 5I). At the same time, the fluorescence intensity of M1 macrophage marker CD86 was significantly increased, while that of M2 macrophage marker CD206 was significantly decreased, indicating that M1 macrophage was activated and its phagocytosis was enhanced (Figure 5J). These results demonstrated that the combination of GOx and GalNAc‐modified LYTACs could significantly inhibit tumor growth in vivo and in vitro by relieving the immunosuppression generated by CD24/Siglec‐10 signal pathway.

## 3 Discussion

In this study, we have developed a lysosome‐targeting chimeric platform based on GalNAc‐modified peptides. The bi‐functional LYTAC molecules can traffic the target membrane protein to the lysosome in a highly specific manner. As seen in the confocal immunofluorescence analysis, the translocation of membrane proteins to lysosomes depends on the binding of Nano‐LYTACs to ASGPR. The degradation of membrane proteins in hepatoma cells can vary greatly depending on the differential expression of ASGPR. The high purification of CD24 scFv ensured efficient linking with nanospheres through crosslinking, and the free carboxyl group in the CD24 antibody could efficiently connect with the free amino group in the nanospheres. Further functional analysis indicated that CD24 degradation mediated by ASGPR‐driven LYTACs affected the signal transmission of the CD24/Siglec‐10 signal pathway, expression of related genes downstream of Siglec‐10 in macrophages, and phagocytosis of tumor cells by macrophages. The results elucidated a crucial role of CD24 in tumor immunosuppression via interaction with Siglec‐10, which acts as a “don't eat me” signal. GOx, an effective tumor starvation therapy drug, has shown excellent therapeutic effects both in vivo and in vitro when combined with Nanosphere‐AntiCD24. We also observed the degradation of CD24 protein by GOx‐LYTACs. To our knowledge, this is the first report to show the function and mechanism of GalNAc‐modified nanospheres in promoting the degradation of membrane proteins.

The elimination of POIs has attracted considerable attention and has broad applications in tumor therapy. Lysosome‐targeting chimeras are an effective method for degrading membrane and extracellular proteins.^[^ ^7^, ^9^ ^]^ Unlike proteolysis‐targeting chimeras, LYTACs take advantage of lysosome targeting receptors such as CI‐M6PR and ASGPR to mediate cargo internalization and shuttle to the lysosome for degradation.^[^ ^41^, ^42^ ^]^ While several vital intracellular targets would benefit from these existing strategies, roughly 40% of protein‐encoding genes are expressed as extracellular/membrane proteins.^[^ ^43^ ^]^ LYTACs for the treatment of liver cancer have witnessed significant progress due to the increased surface expression of ASGPR. Although Ahn et al. pioneered the strategy of using ASGPR for degradation, it was achieved through chemically synthesized small molecules that could not be loaded with drugs. The effect in animals depends entirely on the target of degradation.^[^ ^9^ ^]^ Currently, LYTACs are still in the early stage of development. Many issues still need to be solved, such as enhancing the receptor binding efficiency, applying the method to various antibodies, and conducting effective in vivo experiments.

Based on the strategy of combination therapeutics, we selected GOx, an enzyme involved in glucose metabolism, to mediate glucose deprivation.^[^ ^35^ ^]^ We discovered that GOx, in combination with Nanosphere‐AntiCD24, exhibits synergistic effects at specified concentrations. After treatment, cancer cells succumbed to starvation treatment, and the effect of immunotherapy was enhanced by relieving the immunosuppression induced by the CD24/Siglec‐10 pathway. Moreover, we confirmed the efficacy of the combined treatment strategy using in vitro and in vivo experiments. In summary, we determined the mechanism by which Nanosphere‐AntiCD24 alleviated the immunosuppression caused by tumor cells against macrophages. More importantly, we demonstrated a new treatment strategy in which the combined use of ASGPR‐driven LYTACs and GOx can potentially overcome effector phase immune suppression in the tumor microenvironment.

## 4 Conclusion

In conclusion, a novel ASGPR‐driven LYTACs system was designed and synthesized, which was verified to target POI for translocation to the lysosome and subsequent degradation and drug loading. It is noteworthy that the data could be replicated with other membrane proteins, confirming the impact of CD24 protein degradation in altering tumor immunosuppression. We have shown that the treatment has high efficacy both in vitro and in vivo. Nevertheless, valuable contributions in terms of the ASGPR have been highlighted. The effects of ASGPR‐driven protein degradation warrant further attention in future studies.

## 5 Experimental Section

### Peptide Synthesis and Characterization

The two peptides, Lauryl‐P3GKS and Lauryl‐P3GKS (GalNAc), were further synthesized using solid‐phase peptide synthesis. FITC was conjugated to the hydrophilic lysine residues of the two peptides. All peptides were purified by high‐performance liquid chromatography and characterized by LC‐MS (Bankpeptide, Hefei, China). Peptides with a purity greater than 95% were used for the experiments.

### Particle Size Distribution

Peptide samples were prepared at a final concentration of 100 µg mL^−1^ in Milli‐Q water (Millipore, Shanghai, China), sonicated for 10 min, and used for all measurements. A particle size analyzer (Malvern Panalytical, Shanghai, China) was used to measure the particle size data of the peptides in wet dispersion mode.

### TEM and SEM

TEM samples were prepared on a 200‐mesh copper grid. The peptides were diluted to 10 µg mL^−1^ and used for TEM analysis. Samples were dropped on copper grids, incubated for 5 min, and stained with 2 wt% uranyl acetate for 3 min, following the removal of excess solution. After washing, the samples were air‐dried, and images were obtained using an FEI Tecnai G2 F30 TEM at 200 keV by TEM. SEM was used to study the microstructure of the nanospheres. The samples were prepared by coating them with gold in a sputter coater. The samples were attached to aluminum stubs (32 mm) using double‐backed cellophane tape. The SEM was operated at 3 kV voltage, with a 3.9 mm holder size and 20 mm working distance.

### Cell Lines and Cell Culture

HepG2, Huh7, CHO, and THP1 cells were obtained from ATCC (Manassas, VA, USA). HepG2, Huh7, and CHO cells were grown in Dulbecco's modified Eagle's medium (Gibco, ThermoFisher, Shanghai, China) supplemented with 10% fetal bovine serum (Gibco, ThermoFisher, Shanghai, China). THP1 cells were cultured in Roswell Park Memorial Institute 1640 (Gibco, ThermoFisher, Shanghai, China) medium and supplemented with 10% fetal bovine serum. Unless otherwise specified, all cell cultures were grown in 5% CO_2_, 37 °C.

### Confocal Fluorescence Microscopy Analysis of Protein Shuttle and Degradation

HepG2 cells were seeded in a 12‐well plate at 5 × 10^4^ cells per well with cell‐climbing films (NEST, Wuxi, Jiangsu, China) and allowed to grow overnight. The next day, the medium was discarded, and the cell‐climbing films were washed three times with cold phosphate buffered saline (PBS). The FITC‐labeled nanospheres were added to the plates with further dilution to a final peptide concentration of 10 µg mL^−1^ and incubated at 37 °C for 6 h. Subsequently, the culture medium was discarded, rinsed three times with cold PBS, and the cell membrane dye (ATT Bioquest, Pleasanton, CA, USA) diluted with serum‐free medium was added to cells and incubated for 20 min at 37 °C. The cells were then washed thrice with PBS and fixed with 4% paraformaldehyde for 10 min at room temperature. The cell climbing films were removed and fixed with a fixative containing an anti‐fluorescence quencher (Corning, Shanghai, China). Final images were captured using a two‐photon fluorescence microscope (Nikon, Tokyo, Japan).

The same procedure was used for protein degradation. A lysosome staining probe (Beyotime, Shanghai, China) was added proportionally before cell fixation and incubated for 30 min. The culture medium was discarded, the cells were rinsed three times with cold PBS, and fixed with 4% paraformaldehyde for 10 min at room temperature. The cells were washed thrice with 0.01% PBST (PBS, 0.01% Triton‐100). Cells were blocked with 2% bovine serum albumin (BSA) in PBS for 30 min at room temperature. Anti‐EGFR‐488 (CST, Shanghai, China) diluted with PBS was incubated with the cells for 2 h. Following incubation, the nuclear dye 4′,6‐diamidino‐2‐phenylindole (DAPI , Invitrogen, ThermoFisher, Shanghai, China) was added for 10 min. The cells were washed and photographed subsequently.

### Conjugation of Antibodies

0.4 mg of final concentration of 2 × 10^−3^ m EDC (ThermoFisher, Shanghai, China) was added directly to 10 µL of the final concentration of 2 mg mL^−1^ antibody, which resulted in a tenfold molar excess of EDC to nanospheres. 1.1 mg of final concentration of 5 × 10^−3^ m Sulfo‐NHS (ThermoFisher, Shanghai, China) was added to the reaction. The reaction components were mixed thoroughly and allowed to react for 15 min at room temperature. 1.4 µL of a final concentration of 20 × 10^−3^ m 2‐mercaptoethanol was used to inactivate EDC. The activated nanospheres were separated from excess EDC, EDC by‐products, Sulfo‐NHS, and 2‐mercaptoethanol using a 10 kDa desalting column (Amicon Ultra, Sigma, Shanghai, China) that had been equilibrated with PBS. The separated antibody was re‐dissolved in 500 µL of PBS. Then, 1 mL of GalNAc‐modified nanospheres (1 mg mL^−1^) was added to the solution containing activated antibody, mixed well, and allowed to proceed for 2 h at room temperature. The product was filtered and centrifuged again using a 0.1 µm polyvinylidene fluoride membrane (Ultrafree, Sigma, Shanghai, China) that had been equilibrated with PBS. The antibody concentration was determined using a Pierce BCA Protein Assay Kit (ThermoFisher, Shanghai, China).

### Binding Site Number

Quantification was performed according to Equation (1)

(1) $$N\;=\frac{C_{a}}{C_{n}}\;=\;\frac{\frac{A_{280}\;-A_{\max}\times rf}{21000}}{\frac{A_{\max}}{k}}=\frac{\left(A_{280}\;-A_{\max}\times rf\right)\times k}{21000\;\times A_{\max}}$$

where *N* is the number of IgG antibodies per nanosphere; *C*_a_ and *C*_n_ are the concentrations of IgG attached to the nanospheres and nanospheres in the conjugates, respectively; *A*_280_ and *A*_max_ are the absorbances of the conjugate at 280 nm and *λ*_max_, respectively; and *rf* is the ratio of the absorbance of the nanospheres at 280 nm to that at *λ*_max_. The molar extinction coefficient of the IgG antibody at 280 nm is 210 00 cm^−1^ M^−1^. The *rf* of the 650 nanospheres was 0.347, as shown in Supporting Information Figure S2A. *k* is the slope of the standard curve of the nanospheres generated at a series of known concentrations in M^−1^.

The concentration of IgG antibodies attached to the nanospheres could be calculated according to the Beer–Lambert law as *C* = *A*_280_/*ε*, where the light path length is 1 cm. However, the nanospheres also exhibited absorbance and scattering at 280 nm. To obtain the net absorbance of the IgG antibodies at 280 nm, the contribution of the nanospheres at 280 nm was determined. For the conjugates, the absorbance of the nanospheres at 280 nm was calculated as *A*_max_ × *rf*; *A*_max_ multiplied by *rf*. The concentration of the nanospheres in the conjugates was determined using *A*_max_/*k*. Thus, the number of IgG antibodies attached per nanosphere was determined by the ratio of the concentration of IgG to that of nanospheres in the conjugates.

### siRNA Knockdown

HepG2 and Huh7 hepatocellular carcinoma cells (5 × 10^5^ cells per well in a 6‐well plate) were transfected with siRNA (Sangon Biotech, Shanghai, China) and jetPRIME reagent (4A Biotech, Beijing, China), according to the manufacturer's specifications.

### Protein Extraction and Western Blotting

HepG2 and Huh7 hepatocellular carcinoma cells were seeded at 5 × 10^5^ cells per well in a 6‐well cell culture plate, and 2 mL of medium was added to each well and cultured overnight. Different concentrations of LYTACs were added, and the cells were collected 24 h later; in the time group, 50 × 10^−9^ m LYTACs was added, and the cells were collected at set times. The collected cells were extracted using the total cell protein extraction kit (ThermoFisher, Shanghai, China), and the total protein concentration was determined using the Pierce BCA Protein Assay Kit (ThermoFisher, Shanghai, China). Sodium dodecyl sulfate‐polyacrylamide gel electrophoresis (SDS‐PAGE) (10%) was prepared in advance using the PAGE Gel Fast Preparation Kit (Shanghai Epizyme Biomedical Technology, Shanghai, China), and then the samples were analyzed. EGFR, CD24, NF‐KB, and SOCS3 antibodies (CST, Shanghai, China) were used to detect the corresponding proteins, and *β*‐actin and GAPDH (CST, Shanghai, China) were used as internal references for quantitative analysis. A similar procedure was used for the western blot analysis of the inhibitors.

### Flow Cytometry Analysis

≈2 × 10^6^ HepG2 cells were seeded in 6 cm plates and cultured overnight. The medium was then removed, LYTACs at a final concentration of 50 × 10^−9^ m were added to the fresh medium, and the cells were cultured for another 24 h. The control group was treated with the antibodies. After that, the cells were dyed using the Anti‐EGFR‐488 (CST, Shanghai, China) protocol, and the cells were detected using flow cytometry (Beckman, Shanghai, China). Similarly, HepG2 cells were labeled with red cell membrane dye (CST, Shanghai, China) before co‐culturing for flow cytometry of phagocytosis.

### Protein Purification and Detection

The VH and VL fragments of the CD24 antibody were linked to form the complete variable region gene fragment (scFv). The ScFv gene was cloned into a mammalian expression vector (Shangwei, Shenzhen, China) that could be expressed in CHO cells. CHO cells (6 cm plates up to 80% confluency) were transfected with siRNA (Sangon Biotech, Shanghai, China) and jetPRIME (4A Biotech, Beijing, China) reagent according to the manufacturer's specifications. Total protein was extracted, and His‐tag Purification Resin (Beyotime, Shanghai, China) was added to the lysate. The centrifuge tube was placed at 4 °C and slowly shaken on a shaking table for 2 h to fully bind the target protein with his label. The gel was centrifuged at 1000 *g* for 10 sat 4 °C, washed with PBS four to five times, and the washing solution was retained. The target protein three to four times was eluted, each time with a column volume of the eluent. The eluent was collected at each time point in different centrifuge tubes. The eluent collected was a purified His‐tag protein sample. The purity of the protein samples was tested using Western Blotting.

### In Vivo Stability and Toxicity Analysis

BALB/c mice (female, 6 weeks old) were purchased from Guangdong Medical Laboratory Center, and all mice were intraperitoneally injected with 10 mg kg^−1^ Anti‐CD24 or Nanosphere‐AntiCD24, seven mice per group. The initial grouping of the mice was random, after which no additional randomization or blinding was performed. At the indicated times, blood was sampled from the tail using anti‐coagulant capillary tubes, and serum was separated after centrifugation at 700 *g* for 15 min at 4 °C. Serum antibody levels were assayed by His‐Tag ELISA (Ziker, Shenzhen, China), and liver toxicity was tested using a liver function test reagent (Ziker, Shenzhen, China).

### In Vitro Coculture and Phagocytosis Analysis

THP1 cells were transfected with vectors expressing green fluorescent protein (GFP). GFP+THP1 cells were induced into M1 GFP+THP1 cells with phorbol 12‐myristate 13‐acetate (50 ng mL^−1^) for 24 h, and interferon (20 ng mL^−1^) and lipopolysaccharide (500 ng mL^−1^) for 24 h. HepG2 cells were treated with 50 × 10^−9^ m Nanosphere‐AntiCD24 for 24 h and labeled with red cell membrane dye (ThermoFisher, Shanghai, China) before trypsin digestion. After macrophage adherence, the treated Red+HepG2 cells were co‐cultured with M1 GFP+THP1 cells at a cell ratio of 1:5. To allow for the adherence of macrophages while preventing HepG2 adherence, serum‐free Iscove's modified Dulbecco's medium (IMDM) was placed into plates. Reactions were incubated in a 37 °C incubator. Following incubation, the wells were washed vigorously five times with serum‐free IMDM to wash away nonphagocytosed HepG2 cells. Images were taken at different times, and the supernatant was collected for IL‐6 and TNF‐*α* ELISA detection.

In the same way, HepG2 cells were labeled with pHrodo Red (ThermoFisher, Shanghai, China) as per manufacturer instructions. Briefly, after washing, the cells were replaced the growth medium with Live Cell Imaging Solution. pHrodo was added to the cells at a final concentration of 20–100 µg mL^−1^, and incubate at 37 °C for 5–20 min. The cells were washed with prewarmed, dye‐free medium at pH 7.4 or with Live Cell Imaging Solution. The cells were returned to dye‐free medium at pH 7.4 or Live Cell Imaging Solution, and the cells were imaged using appropriate filters for pHrodo. Nonfluorescently labeled adherent macrophages were cultured with pHrodo‐Red‐labeled HepG2 cells in serum‐free IMDM. Phagocytosis assay plates were placed in a 37 °C incubator and imaged.

### Encapsulate and Characterization of GOx

GalNAc‐modified peptides (1 mg) and GOx (2 mg) were dissolved in 1 mL of water and stirred for 5 min. Particle size distribution, TEM, and SEM were used to characterize the components.

### Cell Viability

HepG2 cells were seeded at 5 × 10^3^ cells per well in 96‐well plates and cultured overnight. The following day, the medium was replaced, and different concentrations of GOx‐LYTACs or antibodies were added. The culture plate was incubated for 48 h, and then 10 µL of CCK‐8 solution (Abcam, Shanghai, China) was added to each well. After incubating for 1–4 h, the absorbance was measured at 450 nm using a microplate reader. Cell viability was calculated using the following formula, and an inhibition curve was constructed. Cell viability (%) = [*A* (dosing)−*A* (blank)]/[*A* (0 dosing)−*A* (blank)] × 100, where *A* (dosing) is the OD value of the wells with cells, CCK‐8 solution, and drugs. *A* (0 dosing): the OD value of the wells with cells and CCK‐8 solution but no drug solution; *A* (blank): the OD of the wells without cells.

### Tumorigenesis Assays in Nude Mice

HepG2, a human hepatocellular carcinoma cell line, was used as the subcutaneous tumor model. Female BALB/c nude mice (6 weeks old) were purchased from Guangdong Medical Laboratory Center, and all mice were injected subcutaneously with 5 × 10^6^ HepG2 cells for tumor growth. After 1 week, when the tumor volume exceeded 50 mm^3^, the mice were randomly assigned to the control, Anti‐CD24, Nanosphere‐AntiCD24, and GOx‐LYTACs groups. The experimental group was injected with 100 µg mL^−1^ Anti‐CD24, Nanosphere‐AntiCD24, and GOx‐LYTACs through the caudal vein once a day for 14 days. The tumor volumes and body weights were measured every 2 days, and continued to be monitored for a week after halting treatment. After collecting blood from the mice via retro‐orbit, the tumors and several organs were removed and fixed in 4% paraformaldehyde. Tissue sectioning, immunohistochemistry, and immunofluorescence experiments of CD86 and CD206 were outsourced to a company (Servicebio, Shenzhen, China).

### Statistical Analysis

Microsoft Excel program or Graphpad Prism 7 was used to calculate the mean ± standard deviation of the sample. For the analysis method, unpaired two‐tailed student's *t*‐tests were used to analyze the differences between the two groups. One‐way analysis of variance followed by Bonferroni's multiple comparison tests was used for multiple comparison test. Statistical significance was defined as ^∗∗∗^ *p* < 0.001; ^∗∗^ *p* < 0.005; ^∗^ *p* < 0.05; n.s. = not significant.

### Declarations—Ethics Approval and Consent to Participate

All animal experiments were conducted according to the guidelines and approval of the Institutional Animal Care and Use Committee of Shenzhen International Graduate School, Tsinghua University, and the Medical Laboratory Animal Center of Guangdong, China (License No. 16 (year 2018)).

## Conflict of Interest

The authors declare no conflict of interest.

## Author Contributions

L.H., S.M., and X.D. conceived, supervised, and acquired funding for the study. K.W., X.D., and L.H. designed the experiments. K.W. performed most of the experiments and data analysis with participation and/or help of X.D., C.Y., Y.H., J.C., and K.L. K.W., X.D., A.Y., S.M., and L.H. wrote and revised the manuscript. All the authors contributed to the discussion during the research and manuscript preparation.

## Supporting information

Supporting Information

## Data Availability

The data that support the findings of this study are available from the corresponding author upon reasonable request.

## References

[1] J. Lin , J. Jin , Y. Shen , L. Zhang , G. Gong , H. Bian , H. Chen , D. G. Nagle , Y. Wu , W. Zhang , X. Luan , Theranostics 2021, 11, 8337. 34373745 10.7150/thno.62686 PMC8344007

[2] K. T. G. Samarasinghe , C. M. Crews , Cell Chem. Biol. 2021, 28, 934. 34004187 10.1016/j.chembiol.2021.04.011 PMC8286327

[3] S. Khan , Y. He , X. Zhang , Y. Yuan , S. Pu , Q. Kong , G. Zheng , D. Zhou , Oncogene 2020, 39, 4909. 32475992 10.1038/s41388-020-1336-y PMC7319888

[4] P. Arora , M. Singh , V. Singh , S. Bhatia , S. Arora , Mini‐Rev. Med. Chem. 2021, 21, 2347. 33634757 10.2174/1389557521666210226150740

[5] G. Ahn , S. M. Banik , C. R. Bertozzi , Cell Chem. Biol. 2021, 28, 1072. 33770486 10.1016/j.chembiol.2021.02.024 PMC8286304

[6] D. Hong , B. Zhou , B. Zhang , H. Ren , L. Zhu , G. Zheng , M. Ge , J. Ge , Eur. J. Med. Chem. 2022, 239, 114533. 35728507 10.1016/j.ejmech.2022.114533

[7] S. M. Banik , K. Pedram , S. Wisnovsky , G. Ahn , N. M. Riley , C. R. Bertozzi , Nature 2020, 584, 291. 32728216 10.1038/s41586-020-2545-9 PMC7727926

[8] B. Ramadas , P. K. Pain , D. Manna , ChemMedChem 2021, 16, 2951. 34296796 10.1002/cmdc.202100393

[9] G. Ahn , S. M. Banik , C. L. Miller , N. M. Riley , J. R. Cochran , C. R. Bertozzi , Nat. Chem. Biol. 2021, 17, 937. 33767387 10.1038/s41589-021-00770-1 PMC8387313

[10] J. Hu , J. Liu , D. Yang , M. Lu , J. Yin , Protein Pept. Lett. 2014, 21, 1025. 24975671 10.2174/0929866521666140626102429

[11] P. P. Breitfeld , C. F. Simmons Jr , G. J. Strous , H. J. Geuze , A. L. Schwartz , Int. Rev. Cytol. 1985, 97, 47. 3000971 10.1016/s0074-7696(08)62348-7

[12] D. Roggenbuck , M. G Mytilinaiou , S. V Lapin , D. Reinhold , K. Conrad , Autoimmun. Highlights 2012, 3, 119. 10.1007/s13317-012-0041-4 PMC4389076 26000135

[13] A. A. D'Souza , P. V. Devarajan , J. Control. Release 2015, 203, 126. 25701309 10.1016/j.jconrel.2015.02.022

[14] X. Huang , J. C. Leroux , B. Castagner , Bioconjug. Chem. 2017, 28, 283. 27966887 10.1021/acs.bioconjchem.6b00651

[15] T. Yamamoto , M. Sawamura , C. Terada , K. Kashiwada , F. Wada , A. Yamayoshi , S. Obika , M. Harada‐Shiba , Nucleosides Nucleotides Nucleic Acids 2020, 39, 109. 31617782 10.1080/15257770.2019.1677911

[16] F. Perrone , E. F. Craparo , M. Cemazar , U. Kamensek , S. E. Drago , B. Dapas , B. Scaggiante , F. Zanconati , D. Bonazza , M. Grassi , N. Truong , G. Pozzato , R. Farra , G. Cavallaro , G. Grassi , J. Control. Release 2021, 330, 1132. 33212117 10.1016/j.jconrel.2020.11.020

[17] D. F. Caianiello , M. Zhang , J. D. Ray , R. A. Howell , J. C. Swartzel , E. M. J. Branham , E. Chirkin , V. R. Sabbasani , A. Z. Gong , D. M. McDonald , V. Muthusamy , D. A. Spiegel , Nat. Chem. Biol. 2021, 17, 947. 34413525 10.1038/s41589-021-00851-1

[18] Y. Zhou , P. Teng , N. T. Montgomery , X. Li , W. Tang , ACS Cent. Sci. 2021, 7, 499. 33791431 10.1021/acscentsci.1c00146 PMC8006166

[19] Y. H. Ni , X. Zhao , W. Wang , Curr. Gene Ther. 2020, 20, 109. 32576128 10.2174/1566523220666200623170738

[20] S. C. Lim , Biomed. Pharmacother. 2005, 59, 351.16087310

[21] S. Lu , Y. Yao , G. Xu , C. Zhou , Y. Zhang , J. Sun , R. Jiang , Q. Shao , Y. Chen , Cell Death Dis. 2018, 9, 646. 29844385 10.1038/s41419-018-0681-z PMC5974417

[22] A. Y. Liu , Y. Cai , Y. Mao , Y. Lin , H. Zheng , T. Wu , Y. Huang , X. Fang , S. Lin , Q. Feng , Z. Huang , T. Yang , Q. Luo , G. Ouyang , Carcinogenesis 2014, 35, 537. 24193512 10.1093/carcin/bgt364

[23] X. Wan , C. Cheng , Q. Shao , Z. Lin , S. Lu , Y. Chen , Tumour Biol. 2016, 37, 6073. 26608371 10.1007/s13277-015-4442-7

[24] L. Li , J. Chen , C. Ge , F. Zhao , T. Chen , H. Tian , J. Li , H. Li , OncoTargets Ther. 2019, 12, 1705. 10.2147/OTT.S196506 PMC6400134 30881025

[25] A. A. Barkal , R. E. Brewer , M. Markovic , M. Kowarsky , S. A. Barkal , B. W. Zaro , V. Krishnan , J. Hatakeyama , O. Dorigo , L. J. Barkal , I. L. Weissman , Nature 2019, 572, 392. 31367043 10.1038/s41586-019-1456-0 PMC6697206

[26] P. Altevogt , M. Sammar , L. Hüser , G. Kristiansen , Int. J. Cancer 2021, 148, 546. 32790899 10.1002/ijc.33249

[27] S. Yin , F. Gao , Front. Immunol. 2020, 11, 1324. 32765491 10.3389/fimmu.2020.01324 PMC7379889

[28] C. A. Bradley , Nat. Rev. Cancer 2019, 19, 541. 31406301 10.1038/s41568-019-0193-x

[29] E. Panagiotou , N. K. Syrigos , A. Charpidou , E. Kotteas , I. A. Vathiotis , J. Pers. Med. 2022, 12, 8. 10.3390/jpm12081235 PMC9409925 36013184

[30] G. Chen , X. Chen , S. King , K. A. Cavassani , J. Cheng , X. Zheng , H. Cao , H. Yu , J. Qu , D. Fang , W. Wu , X. Bai , J. Liu , S. A. Woodiga , C. Chen , L. Sun , C. M. Hogaboam , S. L. Kunkel , P. Zheng , Y. Liu , Nat. Biotechnol. 2011, 29, 428. 21478876 10.1038/nbt.1846 PMC4090080

[31] Q. Wang , D. Niu , J. Shi , L. Wang , ACS Appl. Mater. Interfaces 2021, 13, 11683. 33656325 10.1021/acsami.1c01006

[32] L. Fu , C. Qi , J. Lin , P. Huang , Chem. Soc. Rev. 2018, 47, 6454. 30024579 10.1039/c7cs00891k

[33] Y. Luo , P. Yan , X. Li , J. Hou , Y. Wang , S. Zhou , Biomacromolecules 2021, 22, 4383. 34533297 10.1021/acs.biomac.1c00960

[34] T. He , H. Xu , Y. Zhang , S. Yi , R. Cui , S. Xing , C. Wei , J. Lin , P. Huang , Theranostics 2020, 10, 1544. 32042321 10.7150/thno.40439 PMC6993236

[35] F. Yu , X. Shang , Y. Zhu , H. Lou , Y. Liu , T. Meng , Y. Hong , H. Yuan , F. Hu , Biomaterials 2021, 275, 120927. 34119887 10.1016/j.biomaterials.2021.120927

[36] J. Yu , Z. Wei , Q. Li , F. Wan , Z. Chao , X. Zhang , L. Lin , H. Meng , L. Tian , Adv. Sci. 2021, 8, 2101467. 10.1002/advs.202101467 PMC8498878 34363341

[37] H. Ren , J. Yong , Q. Yang , Z. Yang , Z. Liu , Y. Xu , H. Wang , X. Jiang , W. Miao , X. Li , Acta Pharm. Sin. B 2021, 11, 3244. 34729313 10.1016/j.apsb.2021.05.005 PMC8546854

[38] X. Cheng , Z. Hao , S. Chu , T. Zhang , C. Cong , L. Liu , W. Zhang , J. Gu , S. Ni , D. Wang , D. Gao , Biomater. Sci. 2021, 9, 6116. 34519735 10.1039/d1bm00551k

[39] W. Xie , W. Deng , M. Zan , L. Rao , G. Yu , D. Zhu , W. Wu , B. Chen , L. Ji , L. Chen , K. Liu , S. Guo , H. Huang , W. Zhang , X. Zhao , Y. Yuan , W. Dong , Z. Sun , W. Liu , ACS Nano 2019, 13, 2849. 30803232 10.1021/acsnano.8b03788

[40] H. He , X. Tu , J. Zhang , D. O. Acheampong , L. Ding , Z. Ma , X. Ren , C. Luo , Z. Chen , T. Wang , W. Xie , M. Wang , Immunobiology 2015, 220,1328. 26255089 10.1016/j.imbio.2015.07.010

[41] P. Ghosh , N. M Dahms , S. Kornfeld , Nat. Rev. Mol. Cell Biol. 2003, 4, 202. 12612639 10.1038/nrm1050

[42] M. Spiess , Biochemistry 1990, 29, 10009. 2125488 10.1021/bi00495a001

[43] M. Uhlén , L. Fagerberg , B. M. Hallström , C. Lindskog , P. Oksvold , A. Mardinoglu , Å. Sivertsson , C. Kampf , E. Sjöstedt , A. Asplund , I. Olsson , K. Edlund , E. Lundberg , S. Navani , C. A. Szigyarto , J. Odeberg , D. Djureinovic , J. O. Takanen , S. Hober , T. Alm , P. H. Edqvist , H. Berling , H. Tegel , J. Mulder , J. Rockberg , P. Nilsson , J. M. Schwenk , M. Hamsten , K. von Feilitzen , M. Forsberg , et al., Science 2015, 347, 1260419. 25613900 10.1126/science.1260419

