# Nano‐LYTACs for Degradation of Membrane Proteins and Inhibition of CD24/Siglec‐10 Signaling Pathway

**DOI:** 10.1002/advs.202305364

**Published:** 2023-09-15

**Authors:** Kun Wang, Albert Yu, Kewei Liu, Chunyan Feng, Yibo Hou, Jiawei Chen, Shaohua Ma, Laiqiang Huang, Xiaoyong Dai

*Adv. Sci*. **2023**, *10*, 2300288

DOI: 10.1002/advs.202300288

In the originally published article there are errors in Figure 3D, Figure 4A, and Figure  S3A. The correct Figure 3, Figure 4, and Figure S3 are reproduced below. This errors do not affect the results or conclusions of this article. The authors apologize for any inconvenience this may have caused. This correction has been approved by all co‐authors.

**Figure 3:**
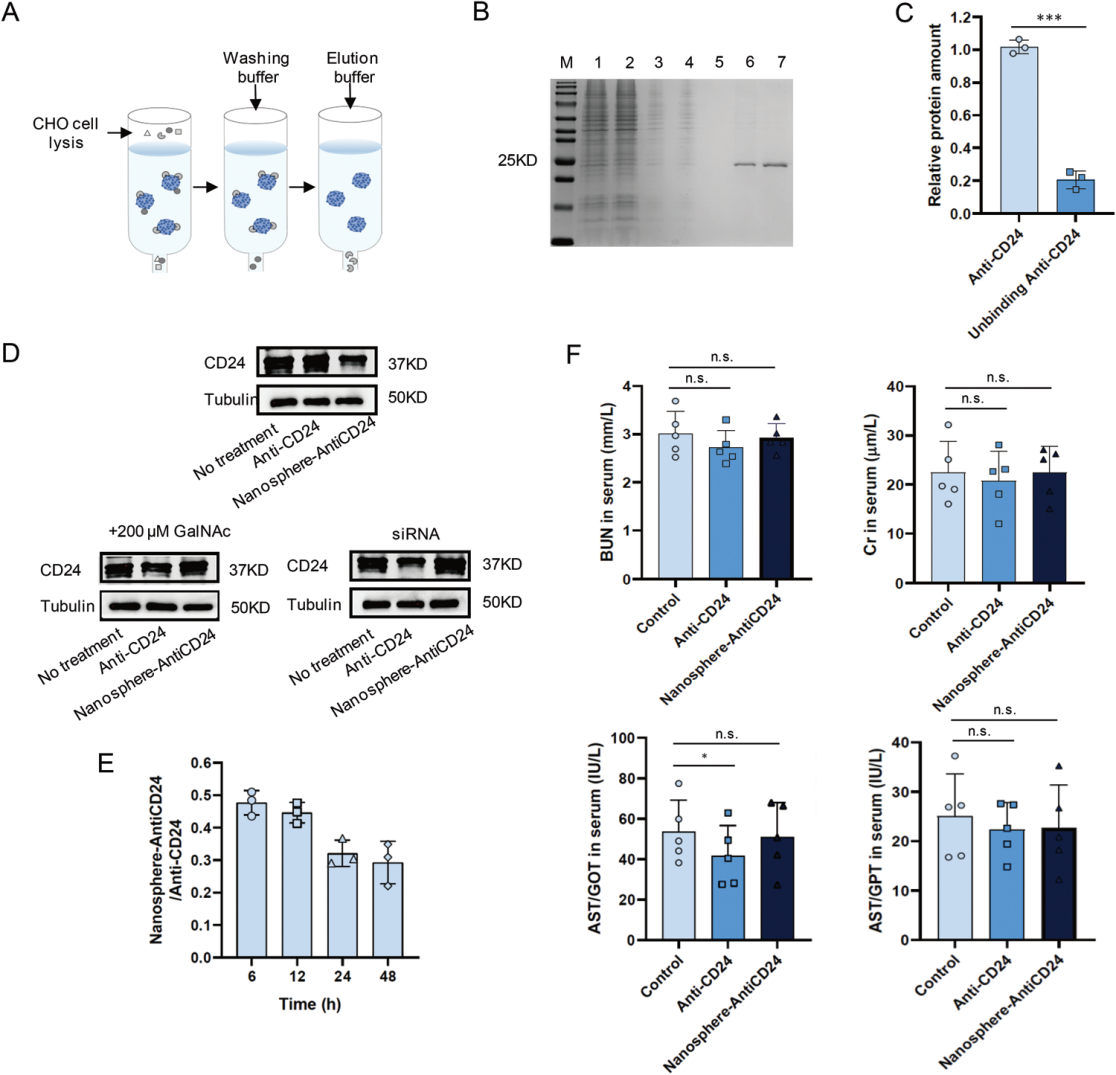
CD24‐targeted LYTACs reinforced degradation of CD24 proteins. A) The protocol of CD24 antibody purification. B) CD24 antibody was analysed by SDS‐PAGE via Coomassie Blue staining (1: cell lysis, 2: unbinding protein, 3: washing 1, 4: washing 2, 5: washing 3, 6: elution 1, 7: elution 2). C) Quantitative detection of Anti‐CD24 protein before and after antibody and nanoparticles conjunction. D) CD24 protein levels after treatment with 100 × 10^−9^ _M_ Nanosphere‐AntiCD24 for 24 h to HepG2 cells in the presence of 200 × 10^−6^ _M_ GalNAc or HepG2 cells expressing a control siRNA targeting ASGPR. E) Quantification of serum Nanosphere‐AntiCD24 relative to Anti‐CD24 after intraperitoneal injection. F) Concentration of aspartic acid transferase (AST), creatinine, and blood urea nitrogen in serum of mice injected with Nanosphere‐AntiCD24.

**Figure 4:**
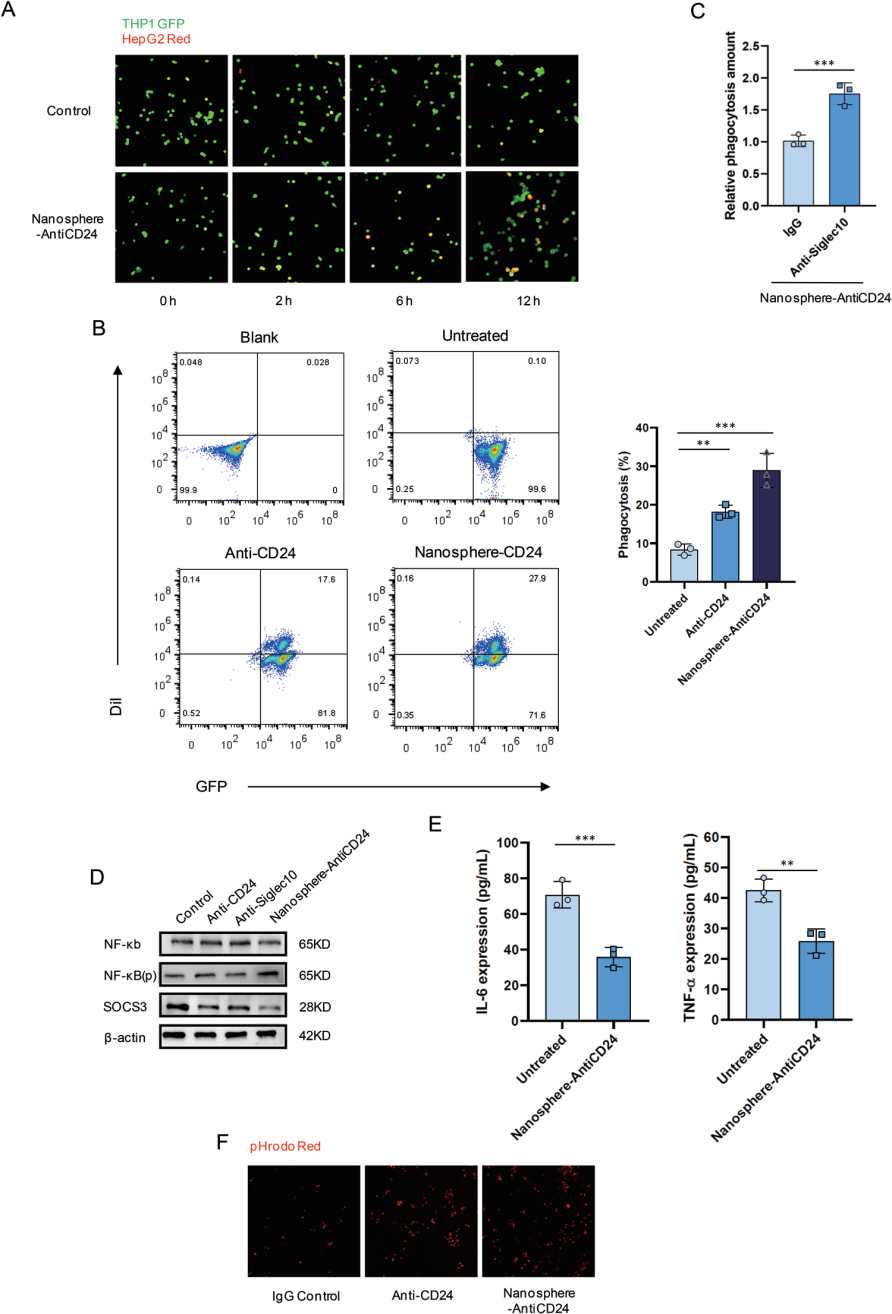
Nanosphere‐AntiCD24 degrades CD24 protein and augments the phagocytic activity of macrophage by blocking CD24/Siglec‐10 signalling pathway. A) Fluorescence microscopy images of the phagocytic activity of THP1 GFP^+^ cells on HepG2 Red^+^ cells treated with 100 × 10^−9^ _M_ NanosphereAntiCD24 for 24 h. B) Flow cytometry‐based measurement of phagocytosis of HepG2 cells (Red^+^) by co‐cultured THP1 GFP^+^ cells, in the presence of 100 × 10−9 m Anti‐CD24 or Nanosphere‐AntiCD24 for 24 h. C) Phagocytosis of HepG2 Red^+^ cells treated with 100 × 10^−9^ _M_ Nanosphere‐AntiCD24 for 24 h in the presence of anti‐Siglec‐10 mAb or IgG control. D) Siglec‐10 downstream NF‐𝜅B, p‐NF‐𝜅B, and SOCS3 levels after treatment with 100 × 10^−9^ _M_ Anti‐CD24, 100 × 10^−9^ _M_ Anti‐Siglec10, or Nanosphere‐AntiCD24 for 24 h. E) Detection of Siglec‐10‐related cytokine secretion. F) Images from live‐cell microscopy phagocytosis assays of pHrodo‐red^+^ HepG2 cells treated with 100 × 10^−9^ _M_ Anti‐CD24, 100 × 10^−9^ _M_ Anti‐Siglec10, or NanosphereAntiCD24 for 2 h.

**Figure S3:**
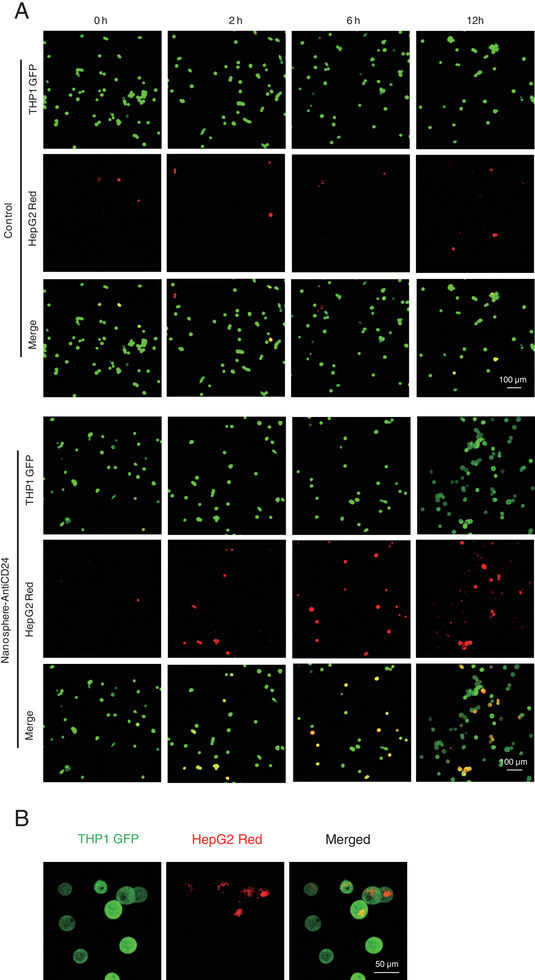
Phagocytosis imaging. A, Single channel fluorescent images of the phagocytic activity of THP1 GFP^+^ cells on HepG2 Red^+^ cells treated with 100 nM Nanosphere‐AntiCD24 for 24 h. B, Representative images collected from high‐resolution confocal fluorescence microscopy of macrophage phagocytosis demonstrating engulfment of whole HepG2 cells (mCherry^+^, Red) by Thp1 (Calcein, AM; green).

