# Supplementary material for: Nano‐LYTACs for Degradation of Membrane Proteins and Inhibition of CD24/Siglec‐10 Signaling Pathway
Source: Adv Sci (Weinh). 2023 Mar 3;10(13):2300288. doi: 10.1002/advs.202300288 (PMC10161071; doi:10.1002/advs.202300288)
Supplement: Supplementary file 1 — Supporting Information [file ADVS-10-2300288-s001.pdf]

ADVANCED SCIENCE
Open Access

Supporting Information

for *Adv. Sci.*, DOI 10.1002/advs.202300288

Nano-LYTACs for Degradation of Membrane Proteins and Inhibition of CD24/Siglec-10 Signaling Pathway

Kun Wang, Albert Yu, Kewei Liu, Chunyan Feng, Yibo Hou, Jiawei Chen, Shaohua Ma, Laiqiang Huang* and Xiaoyong Dai**

Supplementary figures and figure legends

A

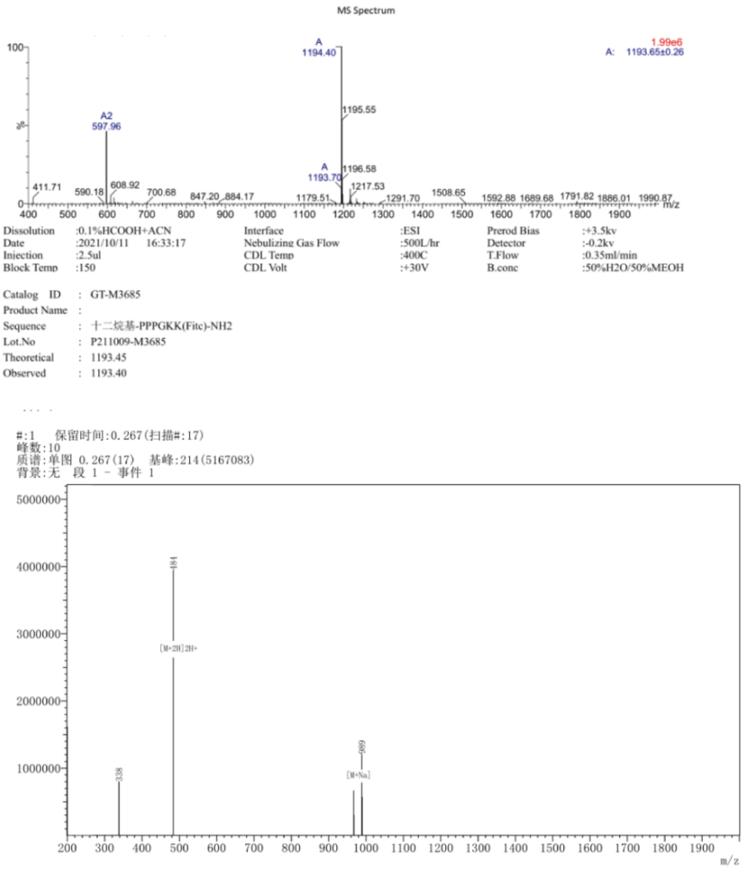

Supplementary Fig. 1. Peptide synthesis detection. **A,** MS detection of glycopeptides, Lauryl-P3GKS and Lauryl-P3GKS (GalNAc).

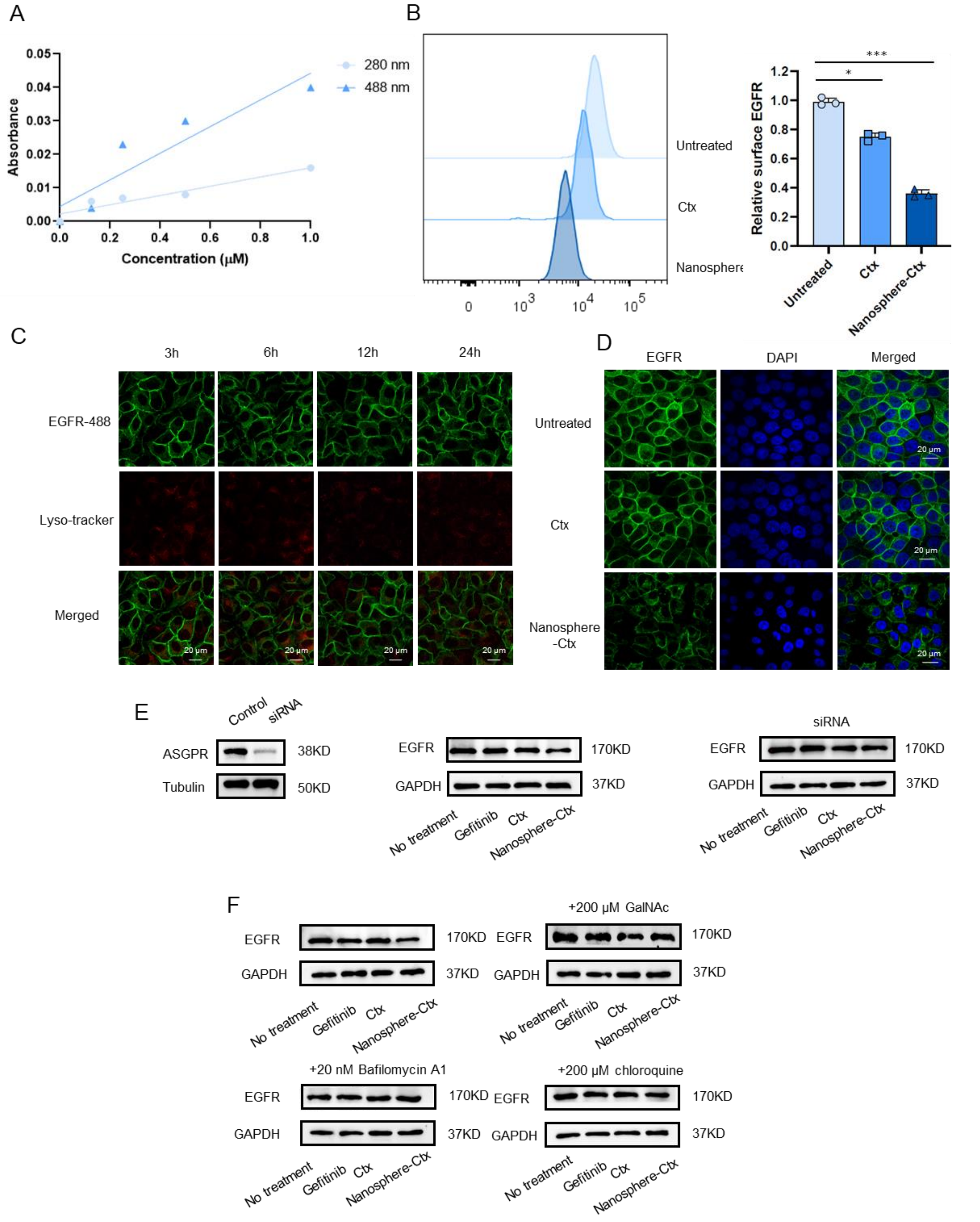

Supplementary Fig. 2. Detection of protein degradation. A, Absorbance detection of antibody conjunction. B, Flow cytometer analysis of EGFR expression on Huh7 cell surface before or after

Nanosphere-Ctx treatment. **C,** Visualization of EGFR re-localization in HepG2 cells by confocal microscopy after treatment with 50 nM Ctx for 24 h**. D,** Visualization of EGFR degradation in Huh7 cells by confocal microscopy after 50 nM Ctx conjugate treatments for 24 h. **E,** EGFR levels after treatment with 50 nM Nanosphere-Ctx for 24 h in Huh7 cells expressing a control siRNA targeting ASGPR. **F,** EGFR levels after treatment with 50 nM Nanosphere-Ctx for 24 h in Huh7 cells in the presence of 200 μM GalNAc, 20 nM BafilomycinA1 or 200 μM chloroquine.

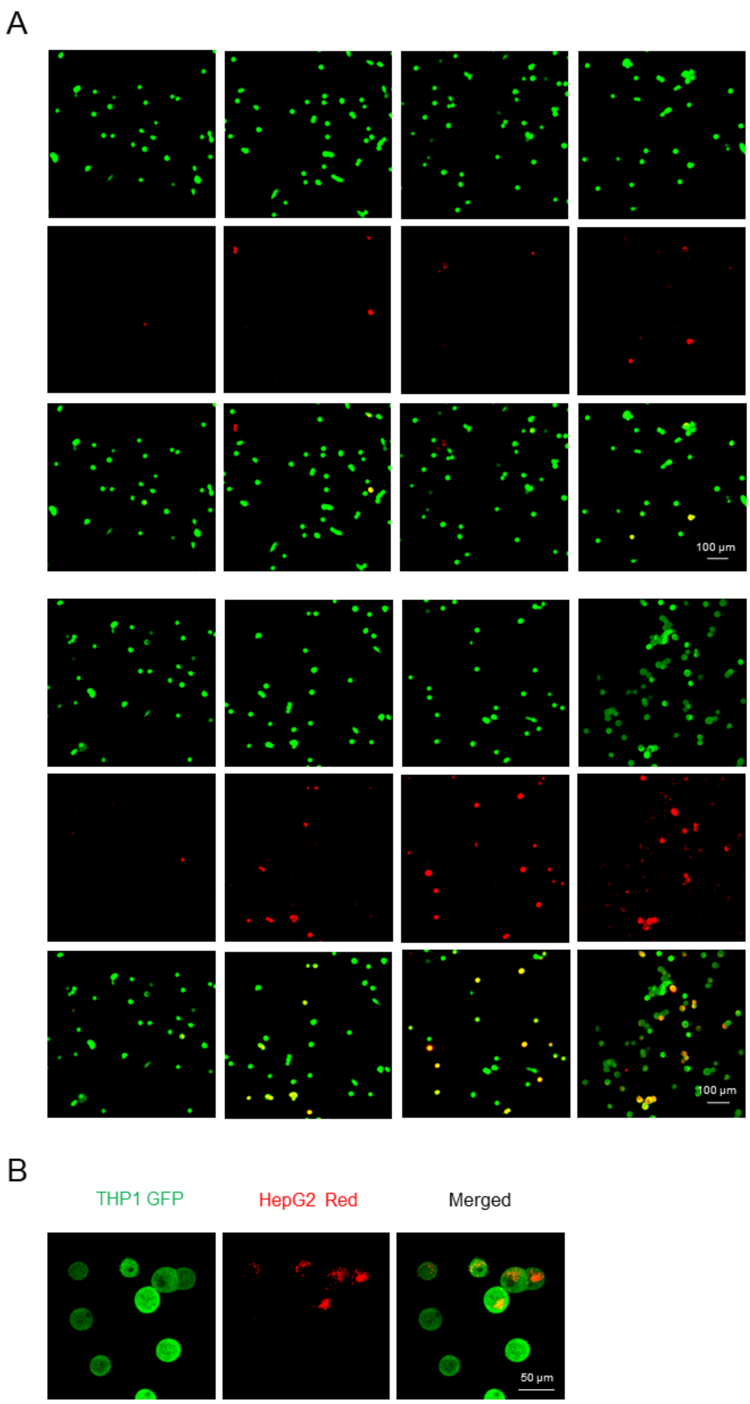

Supplementary Fig. 3. Phagocytosis imaging. **A,** Single channel fluorescent images of the phagocytic activity of THP1 GFP+ cells on HepG2 Red+ cells treated with 100 nM Nanosphere-AntiCD24 for 24 h. **B,** Representative images collected from high-resolution confocal fluorescence microscopy of macrophage phagocytosis demonstrating engulfment of whole HepG2 cells (mCherry+, Red) by Thp1 (Calcein, AM; green).

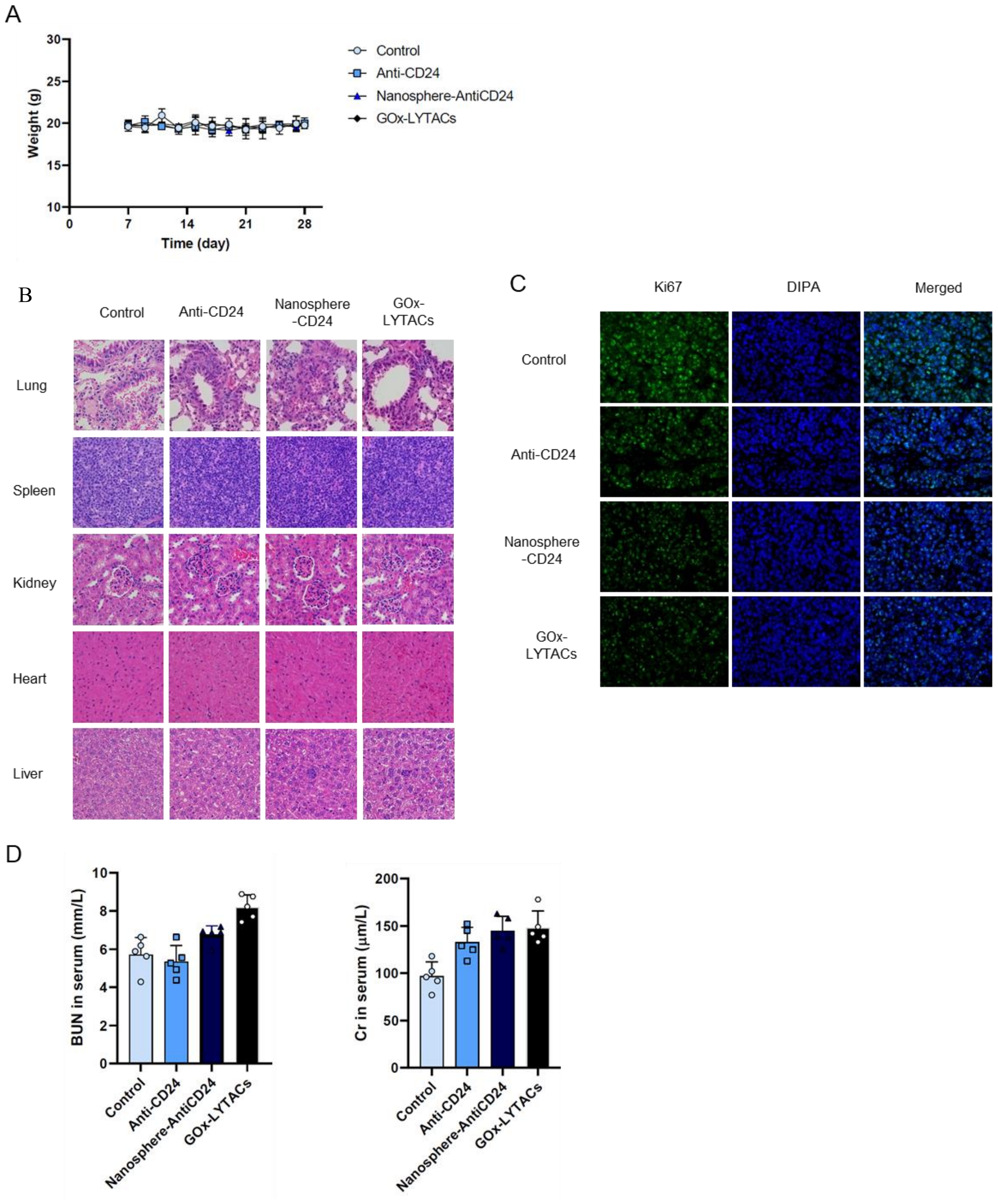

Supplementary Fig. 4. Toxicity test of GOx-LYTACs *in vivo*. **A,** Changes of body weight of mice injected with HepG2 cells. **B,** Immunohistochemistry of Heart, Liver, Spleen, Lung and Kidney treated with 100 ug/kg Anti-CD24, Nanosphere-AntiCD24, GOx-LYTACs for 14 Days. **C,** Immunofluorescence of tumor samples, Ki67 (green) and Cell nuclei (blue). **D,** Concentration of creatinine and Blood urea nitrogen in serum of mice injected with GOx-LYTACs.
